# Implementation of district-based clinical specialist teams in South Africa: Analysing a new role in a transforming system

**DOI:** 10.1186/s12913-018-3377-2

**Published:** 2018-08-03

**Authors:** Kafayat Oboirien, Bronwyn Harris, Jane Goudge, John Eyles

**Affiliations:** Centre for Health Policy, School of Public Health, Faculty of Health Sciences, University of the Witwatersrand, Parktown, Johannesburg, 2193 South Africa

**Keywords:** South Africa, DCST(s), Clinical governance, Implementation, Quality improvement teams, Role, Adaptation

## Abstract

**Background:**

Improving the quality of health care is a national priority in many countries to help reduce unacceptable levels of variation in health system practices, performance and outcomes. In 2012, South Africa introduced district-based clinical specialist teams (DCSTs) to enhance clinical governance at the lowest level of the health system. This paper examines the expectations and responses of local health system actors in the introduction and early implementation of this new DCST role.

**Methods:**

Between 2013 and 2015, we carried out 258 in-depth interviews and three focus group discussions with managers, implementers and intended beneficiaries of the DCST innovation. Data were collected in three districts using a theory of change approach for programme evaluation. We also embarked on role charting through policy document review. Guided by role theory, we analysed data thematically and compared findings across the three districts.

**Results:**

We found role ambiguity and conflict in the implementation of the new DCST role. Individual, organisational and systemic factors influenced actors’ expectations, behaviours, and adjustments to the new clinical governance role. Local contextual factors affected the composition and scope of DCSTs in each site, while leadership and accountability pathways shaped system adaptiveness across all three. Two key contributions emerge; firstly, the responsiveness of the system to an innovation requires time in planning, roll-out, phasing, and monitoring. Secondly, the interconnectedness of quality improvement processes adds complexity to innovation in clinical governance and may influence the (in) effectiveness of service delivery.

**Conclusion:**

Role ambiguity and conflict in the DCST role at a system-wide level suggests the need for effective management of implementation systems. Additionally, improving quality requires anticipating and addressing a shortage of inputs, including financing for additional staff and skills for health care delivery and careful integration of health care policy guidelines.

**Electronic supplementary material:**

The online version of this article (10.1186/s12913-018-3377-2) contains supplementary material, which is available to authorized users.

## Background

Improving the quality of health care is a national priority in many countries to help reduce the unacceptable levels of variation in health system practices, performance and outcomes [1]. Clinical governance is one of the strategies increasingly adopted to address poor quality of healthcare [2, 3]. Clinical governance is a framework or process that guides accountability and establishes specific lines of responsibility for improving clinical practice [2, 4]. Clinical governance seeks to help health care professionals to identify and alleviate error and to promote excellence in the delivery of health care [2]. This involves a range of activities such as risk management, professional development and education, clinical audits, the setting and monitoring of guidelines and standards, and workforce planning [2, 5].

Clinical governance involves multiple actors and this can make implementation unpredictable and challenging (6) especially when lines of responsibility and accountability are not clearly defined [5, 6]. The unpredictability of clinical governance processes are also partly due to the many possible strategies required for promoting good clinical practices. Additionally the range of actors involved in improving the quality of health care may have varying expectations about responsibilities and the behaviour demonstrated when delivering health care might differ from the expected norm [3]. As such, health care providers and managers view clinical governance as punitive and ‘blaming’ especially when there are conflicting variations in expectations [3, 5].

For policy makers seeking to overcome these challenges and improve quality of care, the introduction of new ideas, practices or roles – “innovations” [7, 8] – may strengthen software issues within a system such as lines of responsibility, accountability and interaction [9]. Innovations may be particularly relevant where clinical governance structures and processes are fragmented [10]. For example, the restructuring and development of established teams or the introduction of new teams may reduce professional exclusion and increase the use of system thinking for integrated interaction in quality improvement processes [3, 4]. It is, however, important to understand perceptions and relationships between managers, implementers and intended beneficiaries of clinical governance innovations in order to positively strengthen clinical care. We address this by examining the expectations and responses of local health system actors in the introduction and early implementation of the new clinical governance role of DCST in South Africa’s district health system.

### District-based clinical specialist teams (DCSTs) in South Africa

In 2012, South Africa introduced an innovation to enhance clinical governance and provide supportive supervision in the district health system (DHS): district-based clinical specialist teams (DCSTs) [11, 12]. The DHS is responsible for the management and delivery of primary health care (PHC). Each district has one DCST and each team ideally comprises seven specialists: Obstetrician-Gynaecologist, Advanced Midwife, Paediatrician, Paediatric nurse, Family Physician, PHC nurse and Anaesthetist. This innovation aims to combat South Africa’s poor outcomes in maternal, neonatal, women and child health; improve quality of healthcare; and realise the millennium development goals for mother and child health [13] - 2015 and beyond [14]. Clinical governance is not new in the DHS – the DCSTs were introduced into existing practices, structures and roles that are designed to govern and improve the quality of health care. Given the introduction of the DCSTs into an existing quality improvement environment, we ask: how has the new clinical governance role been perceived and experienced? And why?

We use role theory to examine the expectations, perceptions, and responses of district-based implementers and intended beneficiaries towards the DCSTs. Role theory describes typical behaviour patterns of social and working life (“roles”) [15]. Few studies have used role theory in understanding health service delivery [16, 17], the implementation of health system innovations [18], or how clinical governance works. We engage with these issues in the South African context in light of the introduction and early implementation of the DCST role.

### Roles and milestones in South Africa’s quality improvement programme: Contextualising the new DCST role

**Fig. 1:**
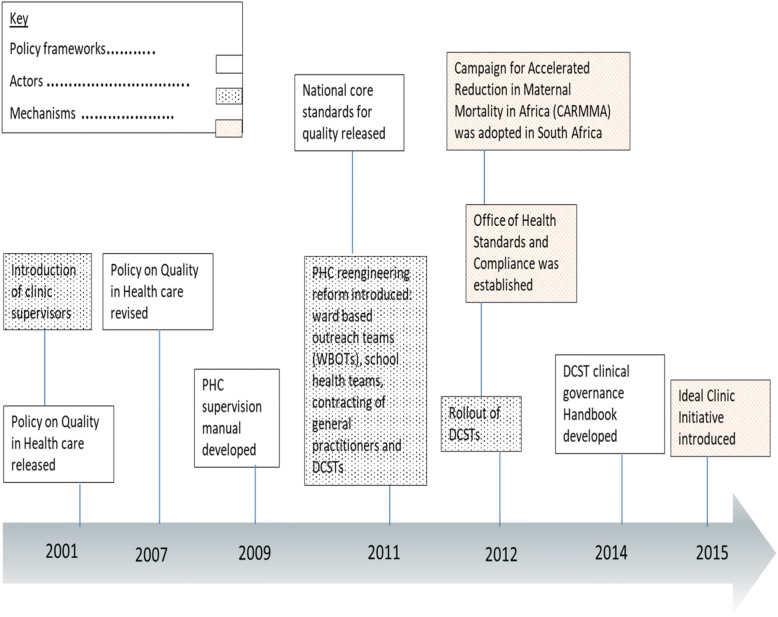
South Africa’s National Quality Policy milestones

In 2001, South Africa set out to create an environment where ‘quality of health care will flourish’ [19]. However, this has not been fully realised. Figure 1 outlines South Africa’s milestones towards quality improvement. Between 2001 and 2015, a number of policy frameworks, actors and mechanisms were introduced. These include the National Quality Policy (2001), revised (2007) which outlines the service delivery and regulatory framework for ensuring access and quality of health care [20]; the PHC supervision manual (2009) [21]; and the National Core Standards for Quality (2011) [22]; and most recently, following the introduction of the DCST (2012), the DCST clinical governance handbook (2014) [23]. All of these quality improvement frameworks aim to provide guidance for assessing quality of health care. Each also led to the introduction of new actors and mechanisms for promoting quality. For example clinic supervisors now known as PHC coordinators, were introduced to implement the PHC supervision manual [21]. In 2012 a regulatory body, the Office of Health Standards and Compliance, was established to drive accountability [20] partly through the newly introduced National Core Standards for Quality [22], which also heralded the DCST clinical governance handbook to guide DCSTs’ activities [23]. Currently, the National Core Standards for Quality are the main standard for assessing care even though existing frameworks or tools are still operational. While old and new quality improvement processes and actors may therefore overlap in policy, this has not been addressed or reported in practice.

### The DCST role

Based on recommendations from a Ministerial Task Team following stakeholder consultations, South Africa’s National Quality Policy framework established the DCST role as:

[The] DCST should ensure quality in clinical services; provide clinical training, monitoring and evaluation, support district-level organisational activities, support health systems and logistics, collaboration, communication and reporting, teaching and research activities [24].

The Ministerial Task Team suggests that, ideally, for administrative matters, members of the DCST will report and account to the district manager, while for clinical matters, they will report to provincial specialists in their respective disciplines [14, 24]. Accountability in clinical supervision is also derived from the National Policy on Quality, which gives all participants (and thereby, all “roles”) within the health care system accountability for improving the quality of health care [19]. Yet the links between different system roles and accountability mechanisms for ensuring quality are not clearly articulated. To the best of our knowledge, nothing has been reported on whether and how quality improvement frameworks, actors and processes influence practices of accountability for supervision and clinical governance in relation to the new DCST role.

### Role theory: A conceptual framework

The health care system is a complex adaptive system because of the multiple, diverse and interconnected parts that make-up the organisation [25]. These system parts are shaped by roles and interactions between roles. Roles are patterns of behaviour and attitudes that an individual exhibits as part of a social or work position and the expectations, which he/she holds for their own behaviour and others in an organisation [26]. Role theory has a long history in social science being developed from the ideas of Mead on self and motive [27] and by Parsons in the 1950s [15]. Role is an important element within an organisation and serves as a basis for identifying and placing persons in a team or organisation [28]. The DCST role is a structural status role, which is a position in a particular organisational setting that forms part of the duties or expected behaviour of individuals [28]. Kahn et al.’s (1964) Role Episode Model [29] as illustrated by Tan (2014) allows for the exploration of relationships between different individuals in relation to a role. This can either involve focal persons (i.e. a person who oversees a particular task, such as a member of the DCST) or as role senders (those who have a stake in the focal person’s role, such as district managers, clinical supervisors and frontline health care workers), both forming a role set [30]. The relationship between role senders and focal persons is interdependent and involves a process of role sending from both sides for a role to be enacted [30]. Table 1 below illustrates some key role concepts relevant to this paper.

**Table 1:** Some relevant role theory concepts and terminologies in the literature

Role concept	Definition	Example
Role differentiation	When we classify given category of individuals with given tasks for the duration of a role system [26]	Distinguishing members of a role set along lines of occupational specialisation (e.g. different specialisation amongst team members in a DCST) or hierarchy (i.e. supervisor and worker line, depending on personal attributes (skills and experience) [26].
Role expectation	What others in the organisation think an individual is responsible for and how the individual should carry out those responsibilities [15, 49].	The role senders’ understanding of the focal person’s job (or vice versa) – based on expected outputs of this person’s role [50]. This may be derived from a work contract, communication by leadership, or policy directives or experiences.
Role behaviour	What an individual actually does in carrying out the job [15, 49].	Both role senders and focal persons exhibit behavior patterns that describe their occupation and reflect the norms and values of the organisation [50].
Role ambiguity	A condition in which expectations or knowledge are insufficient or incomplete to guide behaviour [15].	Due to multiple expectations in an uncertain or complex environment, a focal person may express lack of clarity about how to fulfil demands of the role senders [31].
Role conflict	When there is concurrent appearance of two or more mismatched expectations for the behaviour of a person [15]	Focal persons, whose role spreads across different categories of job interactions, may experience conflicting demands from managers, health professionals or peers [31].
Role consensus	Denotes agreement among expectations that are held by various individuals about a particular role [50]	Ideally, as part of an employment contract, focal persons and role senders are made aware of expected behaviour and rules of enforcement and compliance are agreed upon [50].
Role adaptation/accommodation	A process through which the shared conception and execution of role performance involves flexible combinations of adopted belief, value, coercion and absence of obvious options [28]	Through a change process, a chain reaction results in adjustments to role through a process of diffusion where role begins with a few innovators (focal persons). The process unfurls with more early acceptors, and then an early majority and a late majority, and finally the few laggards. Then diffusion reaches a stage when no more people change to the new role conception [28].

The beliefs and attitudes of individuals about how they should carry out their work can positively or negatively influence how they and others appraise their performance [30]. Each role sending experience is also influenced by how members adjust their expectations [30]. Where expectations differ between focal persons and role senders, role conflicts or ambiguity may emerge, potentially leading to strain on individuals, role sets and the system [15, 31]. When a new role is created, there is role change in the system, which may influence the execution of and adaptation to a role by individuals [28]. Although conflicts and strains may result from variations in expectations, norms, attitudes, and/or behaviours of individuals, other factors may also contribute [15]. These other factors may be contextual and systemic and could include the nature of structural status roles (i.e. positions) within an organisational setting.

In a clinical governance setting, an ideal role set may comprise all health care professionals responsible for ensuring quality in service delivery [2, 5, 32]. In South Africa, DCSTs are seen as focal persons for the governance of quality at the district level. In the DCST policy framework, an ideal clinical governance role set envisages collaboration between DCSTs and a number of potential role senders. These include existing hospital specialists [24], provincial specialists who are DCST mentors [23], members of the District Health Management Team, including PHC and Maternal, Child and Womens’ Health managers, and coordinators at sub-district level, frontline service providers, intersectoral partners, and staff working in other streams of PHC reengineering (ward based outreach teams and school health teams) [14]. In other words, a complex role system has been made potentially more demanding by the creation of DCSTs.

Prior to the implementation of the DCSTs, district-based quality teams and individuals, including clinic supervisors or PHC coordinators (now intended beneficiaries of the DCSTs) were responsible for clinical supervision [21]. While these posts still exist, limited capacity, inadequate operational resources, and irregular monthly supervision visits have been reported as key challenges in different provinces [33, 34]. In addition, limited stewardship and poor management have been documented at all levels of the health system [35].

Given the broad scope of actors and the multiple roles associated with clinical governance, there is a risk of role duplication, conflict, ambiguity or confusion. In this paper, we set out to explore role expectations and behaviours from a range of perspectives, including those of DCST members and their intended beneficiaries. We ask: how has the new clinical governance role [of DCSTs] been perceived and experienced? And why?

## Methods

A theory of change approach to programme evaluation [36, 37] guided our interpretive case study research [38]. Theory of change helps to facilitate direct engagements with and among actors who are part of a change process [36] while utilising a reflective process to construct a shared view of reality through actors’ assumptions, interests and experiences [37]. This involves a logical thinking and action process for understanding the desired change being sought, its context, the process or strategies through which change occurs and factors that influence change [37]. In this study, a theory of change approach allowed participants to reflect on their assumptions, perceptions and experiences of the new DCST role within an existing quality improvement context. It also allowed researchers to construct a logic process of how DCST innovation developed within its context [37] by providing a description of what influences the implementation of the innovation. Theory of change is a critical, participatory approach which is about planning and evaluating practices [36, 37], but also leads to the reflection and possible change in organisational characteristics, including roles. It is thus a transparent methodology for including relevant stakeholder perspectives and thus providing space to co-construct with all participants a collective view of the realities of the DCST innovation.

### Research setting and site selection

This study is part of the UNITAS project (Universal Coverage in Tanzania and South Africa – monitoring and evaluating progress), a five-year study of district-level implementation of innovations towards universal health coverage (2011–2016). In South Africa, we monitored the introduction and early implementation of DCSTs in three of the country’s ten National Health insurance pilot districts [12], using a comparative case study approach. Comparative case studies use two or more cases to identify more generalisable knowledge about causal questions – how and why particular programmes or policies work or fail to work [39]? They involve the analysis and synthesis of similarities, differences and patterns across cases that share a common focus or goal. They are useful for understanding and explaining how context influences the success of an intervention and how better to tailor the intervention to a specific context to achieve intended outcomes [39, 40]. The approach therefore fits well with a theory of change perspective.

South Africa’s DHS is at the base of the national health system, comprising a defined population within a geographical area (a district) with health care facilities serving that population [41]. A district is typically divided into sub-districts and local areas (including clusters of health care facilities – PHC facilities and a district hospital). Each district is managed by; a district health management team and supported by sub-district management teams, PHC managers, PHC coordinators (local area managers or clinic supervisors), programme managers, and facility managers.

Our sampling strategy was informed by an initial context mapping of the ten national pilot districts, based on a review of policy documents and media statements and key informant interviews at a provincial level, where the task of operationalising the national health insurance reforms had been assigned. We then purposefully selected the three districts based on district health management team functionality, the presence of already-recruited DCSTs, and managerial willingness to participate.

At the time of selection, the study sites were (and largely remain) characterised by their poor performance on key health indicators and outcomes [42], including maternal and child health, with maternal mortality and still birth ratios far above the national averages of 133 per 100,000 live births and 22 per 1000 birth respectively [42, 43]. Table 2 presents some differences in the geographical coverage and composition of the DCSTs in the three sites. There are at least three DCST members in each DCST in the three sites but none of the sites has an anaesthetist. This is also observed at a national level where only 9 out of the 49 constituted DCSTs had an anaesthetist [44, 45].

**Table 2:** DCST expected geographical coverage and composition across cases

DCST	District Case 1	District Case 2	District Case 3
Population coverage	1,017,763	695,933	1,364,943
*(Number of fixed facilities)*	*(84)*	*(44)*	*(157)*
Maternal mortality in facility ratio (per 100,000 live births) (2013/14)	208	257	229.7
Stillbirth in facility rate (per 1000 total birth) (2031/14)	32	27	26
Household income (Annual), 2011 ^ZAR^	92, 986	82,266	43,652
Dependency ratio (per 100), 2011	50.7	50.1	80.5
Team complement (composition)	As at March 2015		
Family Physician	○	√	√
PHC Nurse	√	√	√
Paediatrician	X (resigned 2012)	√	√
Paediatric Nurse	√	√	√
Obstetrician & Gynaecologist	X (resigned 2012)	√	X
Advanced midwife	√	√	√
Anaesthetist	X	X	X (resigned 2013)

Key: √ – Filled, x – unfilled, ○ – acting position

Data compiled from: Oboirien et al. 2015 [43]; Census 2011 [43]; District Health Barometer 2013/14 [44] National DCST database, 2015 [45]

Variations between these study sites are seen in the composition of each DCST, population coverage, and geographical terrain (i.e. rural and urban spread). District 1 is the most urban of the three and District 3 is deeply rural. There are also cross-site differences in socio-economic indicators, including household income (higher in the more urban study sites) and dependency ratios (higher in the rural study site). The research took place within the context of provincial moratoria on human resource recruitment in all three study sites, and they were under strain in dealing with human resource turnover and shortages [46].

In each study site, we first carried out a set of in-depth interviews with district-level actors charged with overseeing and managing the implementation of the DCSTs. These interviews helped us to identify where the DCSTs were most active and we then purposefully selected one sub-district in each study site for further in-depth work. At each stage of our inquiry, we used snowballing to identify actors who were interacting with DCSTs; a sampling strategy suitable for the generation of knowledge about a phenomenon (DCSTs) that evolves over time and with inter-related developmental events [47].

### Data collection

Our study approach involved a phased process of engagement at district, sub-district and facility levels, cascading the level of engagement from the implementers to the intended beneficiaries. Data were collected between 2013 and 2015. We carried out 258 in-depth interviews with actors at different levels of the DHS and facilitated one focus group discussion with each DCST (Table 3). A theory of change interview guide (Additional file 1) was used to gather information about the vision and goal of the DCST innovation, expectations about the team and associated activities, assumptions about activities in relation to goals, processes of implementation, actors and relationships needed to drive implementation, and expectations of actor roles in the process.

**Table 3:** Study participants who reflected on DCST roles

Participants	Case 1	Case 2	Case 3	Total
District level - Top management level	23	26	12	61
Sub-district level - Middle management level	4	9	2	15
Facility level - Service delivery level	69	58	55	182
Total number	96	93	69	258

Note: Follow-up interviews are included above

### Data analysis

Our analytical approach involved i. content analysis of policy documents, ii. extracting stories from the reflections of participants, and iii. thematic analysis [40], informed by the iterative process of data collection and engagement with actors, as well as iv. mapping actors who were involved in the implementation of the DCSTs in each district (Additional file 1). We further analysed a “typical” DCST position within the DHS organisational structure using the organisational chart tool in Microsoft Visio 2010. In this actor mapping, we categorised actors into three levels; district level, sub-district level and facility or service delivery level. Then, using policy documents and interviews, we charted roles of expected beneficiaries at the sub-district level/middle management level (those who were involved in some form of clinical governance). Thereafter, we explored the qualitative reflections and experiences of different actors through analysis of the in-depth interviews.

Participants gave informed consent, which was signed and witnessed, and members of the research team carried out interviews in English. Interviews were audio-recorded and transcribed. We coded the transcripts (per participant and by district, sub-district and facility level) manually, using a theory of change data extraction template. Once extracted, we generated codes and developed these into broad themes, capturing and interpreting meaning in this process. We went through stages of data reduction as a crucial part of qualitative analysis [40]. Analysis followed an iterative process of familiarisation with the data, creating themes, and synthesising the data while avoiding over-condensed data (which can lead to loss of content or context) [40]. Interpretation involved situating the DCSTs within the context of each district, as well as the wider national policy discourse, and then inductively locating actors’ reflections within our broader theoretical perspective on roles. Both thematic content analysis (coding and syntheses of different reflections) and extraction of stories (of three DCST members - one in each district) were used to explore the understandings, expectations, and behaviours of different role players about the new DCST role in clinical governance. At least two members of the research team extracted all data and a process of member checking as part of the participant feedback and engagement required of a theory of change method was in place to ensure accuracy in interpretation.

We categorised actor perceptions and experiences into broad themes, for example, position and work experience of actors in DHS, knowledge of the DCST innovation, assumptions about the DCST’s role, activities, and processes. ‘Role’ emerged from the empirical data and was developed into a key analytical idea or ‘sensitising concept’ [48] with a clear definition comprising other attributes or sub-concepts that captured actor accounts of the DCST. Consequently, we turned to role theory as a conceptual framework for informing our analysis and interpretation.

## Results

Using role theory, this section is structured around concepts of role differentiation, adaptation, expectation, and behaviour (Table 1). Role differentiation is applied to how participants distinguish the work of individuals within the clinical governance role set. This concept helps to describe variations in expectations about the DCST role. We also explore the reactions and drivers that influence the ways that actors adjust to this new role (role adaptation) and the understandings and (role) expectations that they, as members of the role set, bring to the DCST role. In addition, the actual activities of members of the role set (role behaviour) are presented as a way of explaining their conceptions of the DCST role.

### Role differentiation - policy versus implementation

We found through our review of the South African health care quality programme (Fig. 1) that there are potential overlaps in the frameworks for assessing quality and actors involved. This is also evident in actors’ perceptions and experiences. In our view, the multiplicity of frameworks and actors within the district health system influenced participants’ expectations and experiences of the DCST role. For example, most of those occupying quality improvement roles at the sub-district level (intended beneficiaries of the DCST) used the National Core Standards for Quality, alongside other supervision tools, to guide their activities. For some, however, these standards were perceived as “independent” from an integrated quality improvement approach [PHC coordinator case 2]. Yet, South Africa’s National Core Standards for Quality framework is supposed to serve as the umbrella policy framework through which roles for quality improvement are derived. Although there was recognition of the need for inclusiveness in quality improvement activities and shared understanding, the central role of the DCST in this process is still unclear among intended beneficiaries due to lack of clarity about the role of DCSTs themselves:

*“…if we ask what the role of the DCSTs is? But it […] won’t filter clearly” [MCWH coordinator case 2].*

### Role differentiation in positions (structural role)

**Fig. 2:**
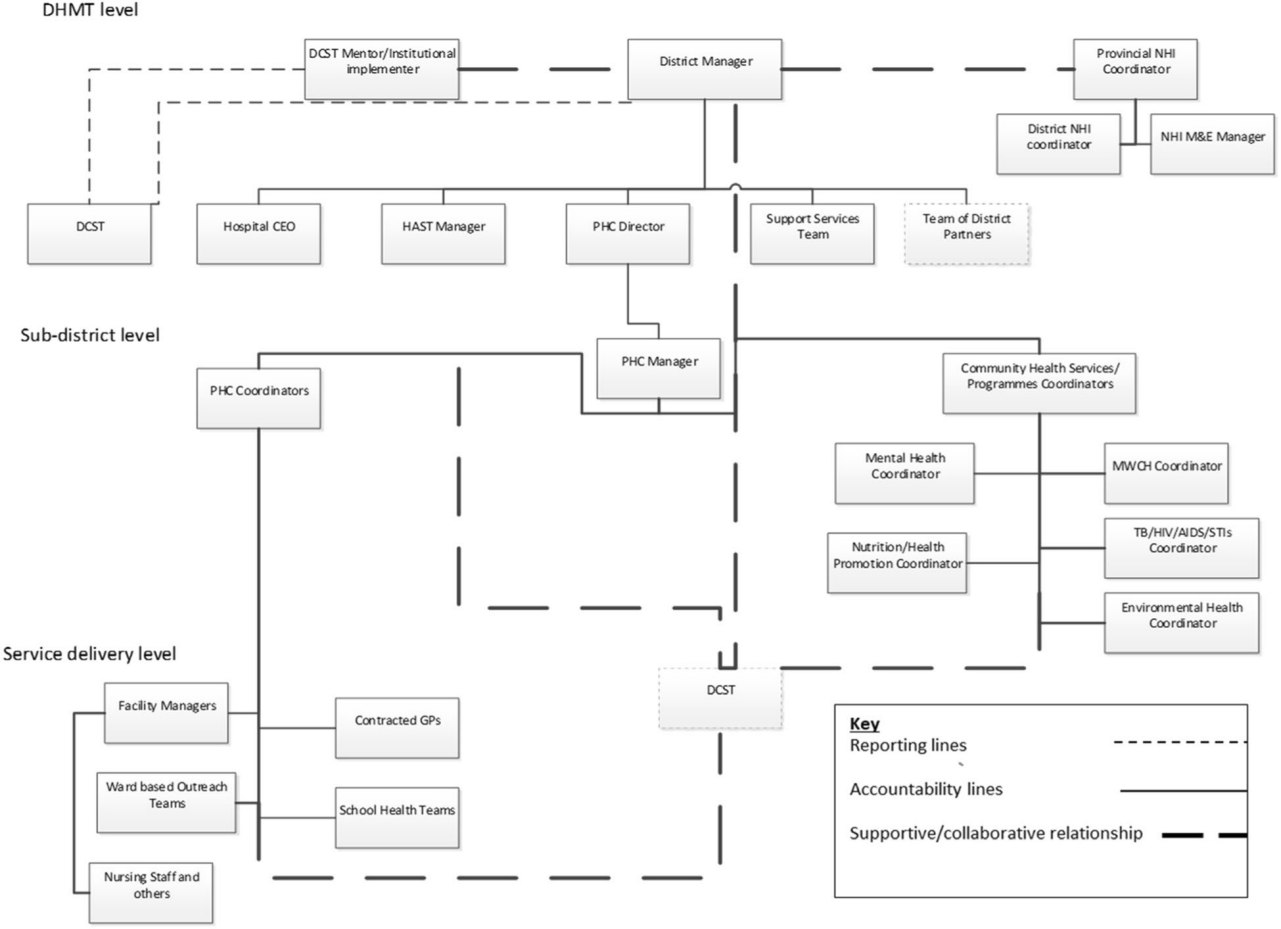
Mapping Actors and Positions in a [typical] South African DHS structure

Figure 2 indicates that the DCST has a uniquely positioned status within the DHS because the team is part of the top district management structure (having access to information on district needs and providing strategic direction) but DCST is also positioned to organise, steer and support processes across the entire DHS structure: a diffuse role without direct authority.

Therefore, structurally, the DCST role brings with it the risk of role ambiguity, confusion, and conflict. As Fig. 2 suggests DCST members report and account to the district manager in all three sites and in policy, they are expected to report to provincial specialists. However, DCST interaction with provincial specialists was not substantially reported during the course of this study. At the same time, no one is directly accountable to the DCST within the DHS structure; rather their structural role is collaborative and supportive. In our study, the potential for collaboration and support was largely dependent on the structural position of those occupying other quality improvement roles. At the top management level, where the DCST structural role is closely linked to that of a wider managerial responsibility for quality improvement, there was a fair level of role consensus. Top managers at the district level in all three districts were clear that the DCST has a role in ‘management’, as well as quality improvement yet they expressed a higher expectation than anticipated by the policy for a DCST role in clinical care and service delivery:

*“…more than being managers or technical advisors, they should assist in clinical care”. [DM Case 3]*

By contrast, role ambiguity and conflict were more often expressed at the sub-district level, particularly around lines of authority and accountability. In all three sites, the DCSTs were perceived to be operating above the authority and jurisdiction of middle managers, as “investigators” [PHC Manager Case 3]. For two sub-district managers, the team was portrayed as by-passing the authority of even the district manager with an imagined reporting line to the National Health Minister. Sub-district managers also raised questions about their own authority in relation to the new DCST role:

*“Somehow DCSTs are infringing into facilities because they want to discipline… as ‘management’ but you won’t come into my house and discipline my child without telling me, so sometimes I feel it is causing friction” [PHC Manager Case 2]*

For those occupying the sub-district tier of the health system, the new DCST role was largely differentiated through hierarchy: less of an advisory/cooperative role (as envisaged in the policy ideal and by the senior district managers) and more of a role that suggests imposed authority. By contrast, some DCST members were uncertain about whether they actually had authority to execute a managerial role.

*“I don’t think that we are managers as such…. We cannot take decisions. We can recommend but what happens after that really is not within our boundaries….” [* ***DCST FGD Case 2]***

While the above reflection suggests that DCST authority was partly constrained by the policy’s expectations of their role, certain DCST members differentiated their role from existing quality improvement actors (such as the PHC coordinators) as less managerial/administrative and more clinical.

*“I am not an implementer but I reinforce implementation of the guidelines but not [in an] administrative role” [DCST case 3].*

This also suggests that while DCST clinical governance role ideally should involve the different forms of role expressed, DCST are articulating the need for a limited role in administrative processes.

### Understanding the path towards role adaptation

#### Context of implementation

Until the introduction of the DCSTs, there was no clearly differentiated or exclusive clinical governance role at the DHS level; hence, the adjustment (by individuals and the system) to this new team is unprecedented. In our study, the district context shaped how intended beneficiaries adjusted to this new DCST role. In particular, the geographical accessibility of District 1 and the deeply rural location of District 3 emerged as important in the recruitment and retention of DCST members. For example, the rural study site saw DCST implementation as a potential source of skilled personnel because *“it’s not easy to attract doctors or specialists” [DM Case 3].*

In all three sites, which struggle with human resource shortages (compounded in each by provincially imposed moratoria on their recruitment strategies), the addition of new specialists was welcomed; an enabling factor in the adjustment to the DCST role. However, in practice, for some intended role beneficiaries, there was a mismatch between actual and expected roles. We use stories of DCST members from each of the three study sites to illustrate the process of adjustment to this new role in clinical governance (Boxes 1, 2 and 3). When read together, they show the interplay between the existing quality improvement system, the history of members of the role sets, the nature of the DCST innovation, and coping mechanisms adopted in the process of adjusting to a new role. These enable the identification of aspects of local context in all three cases.

Story 1 (Box 1) draws attention to a typical DCST approach to clinical governance (observed in all three cases) with DCST activities ranging from identification of system gaps and provision of relevant support (clinical and operational) to management of relationships. This story also reveals challenges of human resource shortages in service delivery (also suffered by all three districts).

#### The context of an existing quality improvement system

A number of constraints emerged as influencing the ‘haphazard’ functioning of the existing quality improvement system which seems to have persisted since the introduction in 2001 of a PHC coordinator role in facility supervision. Firstly, in each of the sites, there was a shortage of PHC coordinators in relation to local needs for facility supervision, as well as transport constraints (in one district), coupled with increasing workload imposed by other national initiatives. For all actors, these constraints set limits on the coverage of supportive supervision at facility level. The perceived limited coverage of PHC coordinators to provide clinical supervision may also be partly due to the multiple, unintegrated supervision tools that were seen as duplicating and time-consuming. For example, quality improvement tools used for the ideal clinic concept and the national core standards for quality are perceived to *“look for the same thing”* but require different forms and assessments *[PHC coordinator case 2*].

Yet, many of the DCST members are of the opinion that middle management actors with quality improvement responsibilities have insufficient clinical capacity. However, this perception was expressed with caution: firstly, because PHC coordinators are seen as enablers to the DCST role and the system, doing *“more than supervision she’s more like a mother to the team” [DCST Case 2]*. Secondly, because some of the DCST members expressed empathy and concern about the constraining environment in which PHC coordinators had to function and felt well positioned to *“influence systems and improve clinical skills” [DCST case 1].*

#### History of role players in the system

Amongst DCST team members, we found (i) those who were new to the district; and (ii) those who had taken up the new role from another post within the district. In all three study sites, most of the DCST members had been employed from within the district.

In story 2, the promotion of a PHC coordinator from within the district into a DCST position (Box 2), reveals the influence of history in shaping expectations and behavior about the new role. Her first-hand knowledge of existing quality improvement activities in the district enabled a quick emergence of role in clinical governance. However, her knowledge of system challenges, as well as relationships between members of the quality improvement role set and their expectations of her, contributed to role conflict and strain.

#### Nature of innovation

The multidisciplinary nature of the DCST and the differences in role across the key specialties (Family Medicine, Paediatrics, Obstetrics and Gynaecology), also influenced the expectations and behaviour of members within the quality improvement role set. For DCST participants in all three districts, there was a fair level of consensus and clarity about the role of those in paediatric and obstetric care; however, the roles of the PHC nurses and Family Physicians were more difficult to understand or link to broader system goals (Box 3). There were expectations that the PHC/Family Medicine roles should inclusively tackle all issues at PHC level, resulting in a slower pace of role adaptation when compared to the other specialties within the DCST:

*“You have a PHC trained Nurse and Physician; what are they doing about other diseases? Diabetes and… Chronic disease…” [PHC coordinator case 2]*

#### Coping mechanisms

Across the study sites, leadership and support from mentors were important in facilitating DCST members’ adjustment to their role. Mentors were found within institutional structures (such as Family Medicine departments, Universities, NGOs), or at an individual level (a senior clinician within the university linked to the district). In their individual and institutional capacities, these mentors played a crucial role in establishing the team, facilitating spaces for DCST members, and providing ongoing support for their integration into the district health system. The story in Box 3 suggests processes used in defining and communicating the new DCST role which assisted team members in clarifying role expectations.

The personalities, communication style, and relationship skills of focal persons within the quality improvement role set also contributed to the promotion of either cooperation or conflict and strain in the role.

*“[Staff at the clinic think] Oh, she’s coming to [look for] wrong things. And when I go inside and greet them with a smile and I introduce myself… then they settle down” [DCST Case 1]*.

An ability to communicate clearly, with willingness to listen to others and a passion to influence change in the system emerged as key traits for successful role adaptation. Furthermore, role players acknowledged the importance of ‘time’, and with this, experience and a growing familiarity with the DCST role, as factors facilitating role adaptation.

## Discussion

### Flexible role boundaries

Our analysis of different actors’ responses and ways of adjusting to the new DCST role reveals continuous processes of role differentiation and role adaptation at different levels of the district health system. Given the potential for complementarity between the DCST and the sub-district level managers, as well as top management levels in quality improvement, this research indicates that a system wide-approach to clinical governance has potential to promote positive change [1]. As visualised in policy, their cross-cutting role positions the DCSTs to implement clinical governance by involving individuals and teams across the district system [1, 3]. In practice, however, the early stage resistance of intended beneficiaries of the DCSTs also highlights certain challenges experienced when introducing an innovation [7]. In addition, roles which have multi-level effects or boundary spanning functions within a system, as is the case of the DCSTs, often expose conflict and stress [31] as a result of competing demands among actors [30, 32].

The range of activities undertaken by DCST members individually and as part of a team reveals clinical, managerial/operational, and administrative dimensions of clinical governance. Performing these dimensions requires professional autonomy and flexibility in the role while also acknowledging the complementarities of members of the role set. Our findings suggest that the flexibility necessary for the DCST role requires some degree of discretion in decision-making but may also influence the perceived dominance of one role (the DCST) over another (for example PHC coordinator) in clinical governance. Having a flexible role in this context may also suggest a tendency to erode the boundaries of related roles. However, as has been noted in other clinical governance settings, the integration between structures and processes is still fragmented [10] including people and the roles they hold.

### Effects of role strain

The role strain expressed in quality improvement processes reveals some of the systemic factors influencing role adaptation, such as human resource shortages and skill gaps and irregularities in practices and routines for supporting service delivery. But, do the strains or conflicts observed in the process of role adaptation lead to beneficial effects or otherwise? Evidence of interpersonal differences and coping mechanisms, leadership, and accountability are central in the process of DCST role adaptation to the benefit of the wider system. Conversely, across the course of our study, accountability concerns emerged in the reflections of actors and shaped role conflict. Here, elements of ‘blame’ emerged, with the PHC managers ‘blaming’ DCST members for not communicating or accounting for their involvement in clinical governance. Such concerns about ‘blame culture’ have also been reported in clinical governance experiences in other settings [3, 5]. Different examples of role sending, challenging of the existing quality improvement system, and adjustment to the new DCST role were seen amongst DCST team members themselves and between DCSTs and others. These processes, expressed through role ambiguity and conflict, reveal challenges within existing quality improvement process. While capacity building and an increase in the number of PHC coordinators may address some of these challenges, role ambiguity and conflict also alert the system to the importance of attending to ‘software’ issues of agency and accountability [9]. Further investigation into the influence of implementation management in integrating roles may thus be useful. In addition, such implementation management processes may form part of a ‘facilitative, developmental and supportive process’ that will encourage a sense of ownership, trust and voluntary engagement for promoting quality [3].

### Feasibility of role consensus amidst alternatives

The limited shared understanding about clinical governance in many settings [6] should be given renewed priority to help reduce ambiguity between leadership, accountability, and integration of roles. In the execution of roles, DCSTs are highly connected to all parts and levels of the district health system, fostering collaboration, exercising leadership, and building capacity through mentorship. The DCSTs’ continuous engagement with other role players is creating spaces for adjustments to role despite the role ambiguity and conflicts observed. However, a comfortable level of role consensus for quality improvement may not be reached, [28] because of the high level of discretion required to undertake clinical governance. Our findings support Turner’s assumption that the most important process in stabilising a (new) role within a system is role adaptation rather than consensus [28]. Even with the high level of consensus about the value added to clinical supervision by the DCSTs, the presence of already-existing quality improvement role-players as apparent alternatives (role of PHC coordinators for example) could challenge role consensus. This further underscores the importance of implementation management systems. Although appointed DCST mentors may be able to partially fill this gap, there is still a need for an integrated approach to clinical governance in different settings. In introducing new roles and in the process of managing human resources for health care, careful consideration for inter/intra role analysis should be factored into the planning, roll-out, phasing, and monitoring of innovations to reduce erroneous and ambiguous expectations about roles.

### The impact of context on role adaptation

By comparing insights across the three sites, we intended to identify some of the contextual factors influencing the implementation of a DCST role. Yet, while the process of role adaptation might be expected to settle more quickly in contexts where DCSTs have a higher team member complement and more resources to operate, in our study, district context did not seem to exert much influence over how actors were adapting to this new role. However, in all three sites, there have been small, yet positive changes in key health indicators overtime despite geographical and human resource constraints (including varying degrees of success with recruiting DCST members in each). For example, between 2013/14 and 2015/16, maternal mortality ratios and stillbirth rates declined marginally in the more urban Districts 1 and 2, than deeply-rural District 3 [42]. However, the link between DCST activities and the observed improvements in some of the maternal and child health indicators have not been established. We point to the importance of a comparative case study approach in helping to highlight this. Therefore, there are implications for role expectations and behaviour given that the extent of DCST coverage is dependent on the composition of the team itself, as well as beneficiaries complementing their work. Future analysis of the DCST’s actual activities may help, to further understand where they are making an impact and how?

It is noteworthy that there was similarity in how intended beneficiaries articulated their expectations of the DCST role in clinical governance although the perceived role conflict observed varied in intensity across sites. In the three study sites, DCST members had similar expectations about what their role should be. This may be due to their standardised induction into the role, a process that was led by the National Department of Health. While often different from DCST members’ expectations, other members of their role set held similar expectations to each other about the DCST role. The differences in expectation and behaviour about the DCST role across sites were not as evident as differences between actors (DCST members versus others). Given the ongoing developments in quality improvement policy and the potential for additional new programmes to target quality, further formative evaluations are important for assessing the receptiveness of the system to change, pointing to the continuing relevance of the theory of change.

### The contribution of a theory of change approach

Methodologically, therefore, the theory of change approach helped actors to reflect on their expectations and experiences of the DCST role in clinical governance. It also provided space for dialogue by bringing together different actors to explore role differentiation. Further, this method influenced the dissemination of stories of positive role adaptation. In our view, sharing of actors’ reflections over time and across sites helped role players to think about how to improve implementation within their local contexts. Theory of change also complements concepts of role theory and how the idea of ‘role’ supports the perspectives and experiences of actors presented in this study. There is bearing in further explanatory research that can further verify the applicability of ‘role’ in other settings to understand other explanatory factors in the implementation of change.

We acknowledge the limitation of not being able to further monitor the DCSTs’ integration process into the district health system due to the study period. In the course of our study, some changes could be observed in DCST role adaptation, particularly a growing sense of normalisation and familiarity with the role, and with this, less ambiguity. However, the passage of time itself will contribute to whether the status quo is reinforced or shifted over a long-term evaluation.

## Conclusion

The implementation of DCSTs into the DHS has broadened South Africa’s ‘specialist’ human resource base at primary care level. This new role offers an opportunity for internal system diagnosis and has potential to support the realisation of global goals such as the MDGs and SDGs [13]. However, our study suggests that the adjustment to the DCST role within an existing quality improvement environment is not necessarily automatic. Rather, problems in policy design may bring role ambiguity and conflict, due to limited clarity about reporting and accountability lines (here, cascading from the DCSTs downwards in the DHS structure). In our study, this was partly explained by the flexible role boundaries in quality improvement, the negative effects of role strain, the difficulty in achieving role consensus due to the apparent alternatives for quality improvement, and the impact of context on role adaptation. Additionally, role conflicts in implementation may have been curtailed if there had been more effective communication between implementing actors. Conversely, processes of role questioning and understanding are crucial for driving system responsiveness to change [28]. We support previous assertions that healthcare organisations should not be seen as machines but as densely connected webs of interacting role players, each operating based on beliefs, local knowledge and interests [25].

The introduction of the DCST role into districts has seen the redirection of specialist roles from a hospital-based approach to an ‘inclusive’ PHC approach. However, PHC as a level of clinical governance within the South African health system is still undergoing transformation. In preparation for the country’s National Health Insurance system, the structure of the district health system is changing. Districts are also responding to innovations and demands because of changing disease patterns and challenges in human resource structure. Improving quality requires anticipating and addressing shortage of inputs for health care delivery and careful integration of healthcare policy guidelines. There is a need for implementation management monitoring that will reinforce the complementary potential of clinical governance within district healthcare. The new clinical governance role of the DCST can help in ensuring quality in service delivery in order for South Africa to move its human resource strength towards universal health coverage.

## Box 1 A day-to-day role

“…As we analyse and support the system in terms of human and material resources...we do get defensive facility managers despite that sometimes we don’t announce our visit and we want to see things as they are (to avoid them getting linen or borrowing equipment for clinical assessment with advance warning). So we just do what we need to do and give the necessary feedback …....We know that existing personnel are trying their best but they are hitting a rock especially in terms of human resources…their functioning is haphazard”. [DCST Case 3]

## Box 2 Changing role within the health system

*I used to be a PHC coordinator [and had to take on the role of coordinating the new ward based outreach team of community health workers] Then I got this DCST post……[But] people automatically had this view that I am moving now to the district to coordinate the ward based outreach teams. That is where the confusion came in… but I stood my ground that I have other responsibilities… [DCST Case 2]*

## Box 3 Clarifying role expectation

*“We had a meeting with role players the CEOs and the HODs, hospitals and MCWH and district team. We were introduced formally and we presented our specific expectations and roles so that they can be clarified. Therefore, that has built most of the relationships so that everybody could feel and see the importance; that we are not here to step in other people’s posts. …. I think that helped a bit… they knew that we are not coming to police them or to find faults”. [DCST Case 1]*

## Additional file

Additional file 1: Question guide for theory of change engagements. (DOCX 97 kb)

## Abbreviations

DCSTs: District-based clinical specialist teams
DHS: District health system
PHC: Primary health care
UNITAS: Universal Health Coverage in Tanzania and South Africa: monitoring and evaluating progress
