# Supplementary material for: Implementation of district-based clinical specialist teams in South Africa: Analysing a new role in a transforming system
Source: BMC Health Serv Res. 2018 Aug 3;18:600. doi: 10.1186/s12913-018-3377-2 (PMC6091061; doi:10.1186/s12913-018-3377-2)

# Additional file 1

## Table S1:

| **Theory of change question guide for individual and focus group discussions** |
| --- |
| - Can you explain your position and work experience in the district? What is your role and who do you report to? - What is your overall vision or goal for this policy or innovation (e.g. DCSTs) and how was it introduced to you? - Do you believe this is a rationale goal to work towards and why? - What are the key activities you will implement to achieve that goal? - What assumptions underpin the selection of these activities in relation to the vision or goal? Why do you believe these activities are key activities? - How will you go about implementing these activities? - What are the most important relationships between these activities and why is the relationship important? - Who will be involved in implementing the activities that will help achieve your goal or vision? - What assumptions underpin the role of actors you will involve and what are the expectations you have of their role in taking action towards your vision or goal? |

## Figure S1:


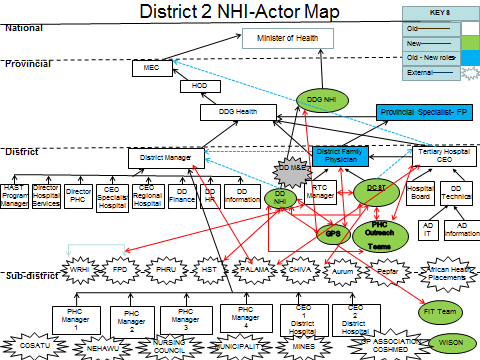


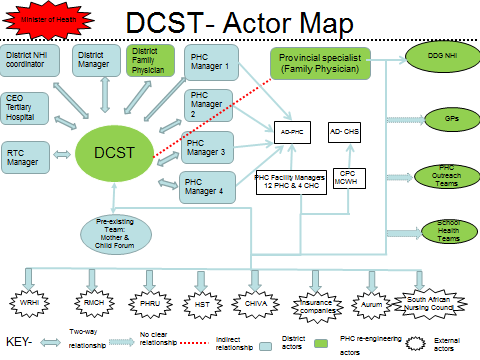

Supplement: Supplementary file 1 — Question guide for theory of change engagements. (DOCX 97 kb) [file 12913_2018_3377_MOESM1_ESM.docx]
